# Age-of-onset-dependent influence of *NOD2* gene variants on disease behaviour and treatment in Crohn’s disease

**DOI:** 10.1186/1471-230X-13-77

**Published:** 2013-05-02

**Authors:** Carsten Posovszky, Veronika Pfalzer, Georgia Lahr, Jan Hendrik Niess, Jochen Klaus, Benjamin Mayer, Klaus-Michael Debatin, Georg BT von Boyen

**Affiliations:** 1 Department of Pediatrics and Adolescent Medicine, University Medical Center Ulm, Eythstr. 24, Ulm, 89075, Germany; 2 Internal Medicine, University Medical Center Ulm, Albert Einstein Allee 24, Ulm, 89081, Germany; 3 Medical department of the Academic Clinic Sigmaringen, Hohenzollernstr. 40, Sigmaringen, 72488, Germany; 4 Institute of Epidemiology and Medical Biometry, Ulm University, Ulm, Germany

**Keywords:** Crohn’s disease, NOD2, CARD 15, Osteoporosis, Pediatric-onset

## Abstract

**Background:**

Influence of genetic variants in the *NOD2* gene may play a more important role in disease activity, behaviour and treatment of pediatric- than adult-onset Crohn’s disease (CD).

**Methods:**

85 pediatric- and 117 adult-onset CD patients were tested for the three main *NOD2* CD-associated variants (p.R702W, p.G908R and p.10007fs) and clinical data of at least two years of follow-up were compared regarding disease behaviour and activity, response to therapy and bone mineral density (BMD).

**Results:**

Chronic active and moderate to severe course of CD is associated in patients with pediatric-onset (p=0.0001) and *NOD2* variant alleles (p=0.0001). In pediatric-onset CD the average PCDAI-Score was significantly higher in patients carrying *NOD2* variants (p=0.0008). In addition, underweight during course of the disease (p=0.012) was associated with *NOD2* variants. Interestingly, osteoporosis was found more frequently in patients carrying *NOD2* variant alleles (p=0.033), especially in pediatric-onset CD patients with homozygous *NOD2* variants (p=0.037). Accordingly, low BMD in pediatric-onset CD is associated with a higher PCDAI (p=0.0092), chronic active disease (p=0.0148), underweight at diagnosis (p=0.0271) and during follow-up (p=0.0109). Furthermore, pediatric-onset CD patients with *NOD2* variants are more frequently steroid-dependent or refractory (p=0.048) and need long-term immunosuppressive therapy (p=0.0213).

**Conclusions:**

These data suggests that the presence of any of the main *NOD2* variants in CD is associated with osteoporosis and an age of onset dependent influence towards underweight, higher disease activity and a more intensive immunosuppressive therapy. This observation supports the idea for an early intensive treatment strategy in children and adolescent CD patients with *NOD2* gene variants.

## Background

Inflammatory bowel disease (IBD) is characterized by chronic inflammation of the gastrointestinal tract and can occur at any age. Crohn’s disease (CD), ulcerative colitis (UC) and indeterminated colitis are defined according to the clinical, endoscopic, radiological and histopathological features. IBD is a multifactorial disease caused by genetic, bacterial, nutritional, and immunological factors. There is clear evidence for a strong genetic influence in the pathogenesis of IBD, especially in CD, coming from twin studies [1] with disease concordance up to 50% for monozygotic CD twins. In addition, pediatric-onset CD is characterized by distinct phenotypic differences compared to adult-onset suggesting that a genetic susceptibility may play an important role [2]. Polymorphisms in the leucine-rich repeats (LRR) region of the *NOD2/CARD15* gene were identified as independent risk factors for CD in Caucasians [3,4], which varies depending on the number of mutations from a 2 to 4-fold increased risk with 1 of the 3 major alleles to 40-fold increase in homozygous or compound heterozygous carriers [5]. The three major polymorphisms in the coding region of *NOD2/CARD15*, associated with CD, are p.R702W, p.G908R, and p.L1007fs. The *NOD2* gene is involved in the innate immune response by recognizing muramyl dipeptide of intracellular bacterial lipopolysaccharides and activating nuclear factor κB pathways [6]. NOD2 is mainly expressed in Paneth cells in the small bowel and is involved in the innate immune response to bacterial pathogens [7]. Thus variant *NOD2* alleles are associated with reduced defensin expression in response to bacteria [8]. Children have shorter environmental exposure and therefore genetic factors such as *NOD2* variants may have a greater influence in the etiology and severity of CD [9]. Disease behaviour may change over time, thus long-term follow-up data on pediatric-onset disease are needed [10]. Indeed, the few studies analyzing genotype-phenotype in pediatric-onset CD with longer observation time revealed associations of *NOD2* variants with disease severity and risk for surgery [11,12].

Children and adolescents suffering from IBD are at risk of developing osteoporosis. The prevalence of severe demineralization in children and in adults ranges from 4.35% to 42% [13]. The pathogenesis of osteoporosis in CD is not completely understood. Bone mineral density (BMD) is influenced by several factors including chronic or recurrent corticosteroid use, malnutrition or malabsorption, low body mass index (BMI), hypogonadism, immobilisation, vitamin D deficiency [14]. Together these factors result in low turnover bone loss. However, also inflammation may significantly contribute to a high-turnover mineral bone loss in CD [14-16]. Osteoclastogenesis and bone resorption is driven by inflammatory mediators such as tumor necrosis factor (TNF) α, Interleukin (IL)-1β and Il-6 in synergy with receptor activator of nuclear factor kappa B ligand (RANKL). RANKL is released by activated T-cells and binds RANK on osteoclastic precursors and thereby induce and activate osteoclasts [17]. Thus a chronic inflammatory state, such as the gut in CD patients, in addition affects bone remodelling and contributes to high turn-over mineral bone loss. The influence of NOD2 genotype on osteoporosis in CD has not been studied yet.

We therefore wished to investigate the potential relationship between the three major *NOD2/CARD15* variants in a well characterized, long term follow up dataset of Crohn patients with pediatric- and adult-onset and inflammatory phenotype, response to therapy and lower bone mineral density.

## Methods

### Study population and disease phenotype

The single center study, approved by the Ethics committee Board of the Ulm University, adheres to the ethical principles of the Helsinki Declaration. 202 patients were recruited in the department of pediatrics and adolescent medicine as well as in the department of internal medicine at the Medical University Center Ulm. The patients or their parents gave written informed consent to participate in the study.

The patient’s diagnosis based on clinical, radiological, endoscopic and histopathologic findings, and presence of disease for at least 2 years from diagnosis with follow-up. Patients with uncertain diagnosis or unclassified colonic inflammatory bowel disease were excluded. The pediatric-onset cohort included patients with age of onset until the age of 18 and final diagnosis until the age of 19. Parameters including age of diagnosis and duration of disease, sex, disease localization, disease behaviour, family history of IBD, body weight and length, body mass index (BMI), extraintestinal involvement, bone mineral density, medical and surgical therapy and disease activity including hospitalization were updated, analyzed and classified according to the Montreal classification. Disease location and behaviour was defined as involvement at any time during disease. Underweight was defined as BMI below 10^th^ percentile for age. Growth failure was defined as inappropriate growth velocity for age and short stature was defined as height below the 3^rd^ percentile for age on a growth chart. Disease activity was calculated based on PCDAI for children with Crohn’s disease [18] or CDAI for adults during regular or emergency visits and given as mean value. An average PCDAI/CDAI score above ≥ 10/150 was defined as chronic active and ≥ 30/220 as moderate to severe disease. In children, a hospitalization of more than two weeks per year due to CD was given to describe disease activity and the percentage of severe ill patients.

### Bone mineral density measurement

Bone mineral density of the lumbar spine (L2-L4) was evaluated using dual-energy X-ray absorptiometry (DEXA) in patients with inflammatory bowel disease using a Hologic QDR 1000 and expressed as grams per square centimetre. Osteoporosis was defined in children as less than two standard deviations (SD) of BMD adjusted to an age matched population (Z-score) [19] and in adults as BMD compared to a young normal reference mean below 2.5 SD (T-score) [20].

### Therapy modalities

The treatment was according to the German guidelines [21] and blinded to the genotype data. Patients received prednisolon (2 mg/kg up to 100 mg/d), budenoside (9 mg/d), azathioprine (AZA) (2.0-2.5 mg/kg/d), 6-mercaptopurin (6-MP) (1–1.5 mg/kg/d), methotrexate (MTX) (15 mg/m^2^/week s.c.), infliximab, a chimeric monoclonal antibody to TNFα (5 mg/kg at weeks 0, 2, 6 and every 8 weeks), or adalimumab, a humanized monoclonal antibody to TNFα (80 mg starting dose, followed by 40 mg every 8 weeks in adults or dose-adjusted for children 24 mg/m^2^ and adapted for through level). Patients with chronic active disease who did not respond to steroid therapy or relapsed within the first 4 weeks were defined as steroid-dependent or steroid-refractory respectively. These patients were then switched to an immunosuppressive long-term therapy with AZA, 6-MP, MTX or TNF-α antagonists. Patients with pediatric-onset received TNF-α antagonists as second line therapy if enteral therapy, AZA, 6-MP or MTX failed to induce stable remission, withdrawal of steroid treatment was impossible and surgical treatment was not reasonable or not accepted by the patients or their guardians.

### DNA extraction and genotyping of NOD2 variants

Genotyping of the three main *NOD2* variants p.R702W (rs2066844, exon 4), p.G908R (rs2066847, exon 8), and p.1007fs (rs20066847, exon 11) were performed in 85 pediatric and 117 adult CD patients of Caucasian origin. Therefore peripheral blood mononuclear cells (PBMCs) of the patients were obtained. Genomic deoxyribonucleic acid (DNA) was extracted from PBMCs using standard methods using QIAmp Flexi Gen DNA Kit (Qiagen, Hilden, Germany). Exons 4, 8 and 11 of the *NOD2* gene were individually amplified by polymerase chain reaction (PCR) analysis using standard methods. PCR-samples were sequenced using the CEQ Quick Start Kit on a GeXP Genetic Analysis System (Beckman Coulter, Krefeld) and compared with wild-type *NOD2*-gene sequence.

### Statistical analysis

Absolute and relative frequencies were used to describe qualitative variables descriptively, and mean with standard deviation as well as median with interquartile range for continuous variables. Differences between frequencies of qualitative characteristics in CD patients carrying *NOD2* variants or wild-type were compared using chi-square test or Fishers exact test, respectively. Mann–Whitney or Kruskal-Wallis tests were applied to compare medians and T-test to compare means, respectively. A *p* value ≤ 0.05 was considered to be significant. Descriptive statistics for quantitative values were given as median and interquartile range in box plot diagrams using Graphpad Prism software. All statistical tests are interpreted explorative. To adjust for possible confounding, multivariate regression analyses have been conducted in the course of a sensitivity analysis.

## Results

### Demographic and disease characteristics of the study population

**Figure 1:**
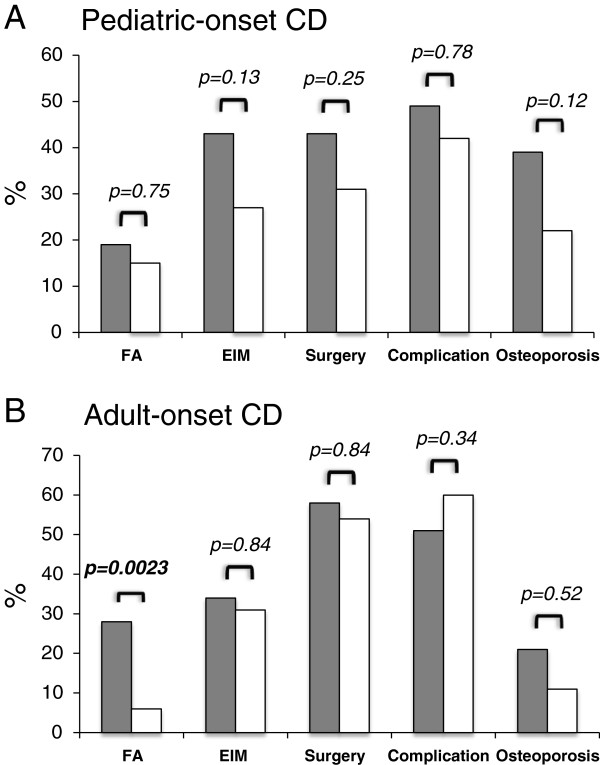
**Effect of *NOD2 *gene variants in CD patients on disease behaviour.** CD patients with pediatric-onset (**A**) or adult-onset (**B**) of disease with *NOD2* variant (grey bars) or *NOD2* wild-type alleles (white bars) are compared regarding family history (FA) of IBD, extraintestinal manifestation (EIM), surgery or complication because of CD, and osteoporosis. BMD was available from 169 out of 202 patients. Differences were compared using chi-square or Fishers exact test, respectively. A p-value below ≤0.05 was considered to be significant.

Data regarding *NOD2* genotype, age of onset, average disease duration, disease location and behaviour were obtained from two hundred and two German patients with adequate follow-up and are presented in Table 1 according to the age of onset of CD. *NOD2* variant alleles were found in 37 out of 85 pediatric- (44%) and 47 out of 117 adult- (40%) onset CD patients (Table 1). No significant differences were noted in the distribution of the three main *NOD2* allele variants (data not shown), however the presence of two variant alleles trend to be higher in pediatric-onset than in adult-onset patients (p=0.12) (Table 1). The mean follow-up was around 11 years in the pediatric-onset group and 16 years in the adult-onset group. Interestingly, the prevalence of IBD in first- or second-degree relatives of CD patients is statistically more frequent in adult-onset CD patients with *NOD2* variants in contrast to *NOD2* wild-type (29% versus 6%; p=0.0023) but not in the pediatric-onset CD patients (Figure 1).

**Table 1:** **Characteristics of 202 patients with pediatric- and adult-onset Crohn’s disease according to the Montreal classification**[22]**regarding *NOD2 *genotype at latest follow-up**

**Patients**	**Pediatric-onset CD (≤18 y)**			**Adult-onset CD (>18 y)**		
**n=202**	**n=85**			**n=117**		
***NOD2 *genotype**	**Main variants**	**Wild-type**	**p-value**	**Main variants**	**Wild-type**	**p-value**
**Number: n (%)**	**37 (44%)**	**48 (56%)**		**47 (40%)**	**70 (60%)**	0.67
1 variant allele	23 (27%)			37 (32%)		
2 variant alleles	14 (16%)			10 (9%)		0.12
homozygous variant alleles	7 (8%)			3 (3%)		0.099
**Gender: female/male (%)**	57/43	38/62	0.08	51/49	59/41	0.39
**Age: year**						
mean/SD	26/13.0	22.7/12.6	0.26	47.3/14.3	46.5/13.4	0.67
median/interquartile range	22/17-35	20/15-26	0.18	46/36-62	45/36-54	0.78
average disease duration/SD	12.8/11.2	11.3/10.5	0.63	16.2/11.3	16.5/10.3	0.89
**Age at diagnosis: year**						
mean/SD	13.9/3.3	12.1/4.9	0.07	31.5/10.0	30.5/10.6	0.49
median/interquartile range	15.0/12-17	13.0/9-17	0.18	27/23-33	29/24-38	0.32
**Localization: n (%)**						
L1 ileal	9 (24%)	12 (25%)	1.0	18 (38%)	14 (21%)	0.058
L2 colonic	3 (8%)	13 (27%)	0.048*	4 (8%)	11 (16%)	0.27
L3 ileocolonic	26 (68%)	23 (48%)	0.048*	25 (53%)	41 (61%)	0.35
L4 isolated upper disease	0 (0%)	0 (0%)	1.0	0 (0%)	1 (1%)	1.0
Upper GI involvement	12 (32%)	16 (33%)	1.0	1 (2%)	7 (10%)	0.14
**Behaviour: n (%)**						
B1 non-stricturing/penetrating	16 (43%)	33 (69%)	0.026*	16 (35%)	28 (41%)	0.53
B2 stricturing	15 (41%)	6 (13%)	0.005*	22 (48%)	20 (29%)	0.051
B3 penetrating intern	6 (16%)	3 (6%)	0.16	12 (26%)	11 (16%)	0.23
B3p penetrating perianal	7 (19%)	7 (15%)	0.76	8 (17%)	19 (28%)	0.27

* indicates p value ≤ 0.05 considered to be significant.

Carrying at least one *NOD2* variant allele is associated with an ileocolonic (L3) localization (p=0.048) in the pediatric- and a trend to an ileal involvement alone in the adult-onset CD patients (38% versus 21%; p=0.06), while pediatric-onset *NOD2* wild-type patients more frequently had a colonic involvement alone (p=0.048). The influence of *NOD2* variants towards complicated disease behaviour (B2 or B3) is more pronounced in patients with pediatric-onset CD (p=0.026). Stricturing disease behaviour (B2) is significantly associated with *NOD2* variant alleles in pediatric-onset (p=0.005) and adult-onset CD Patients (p=0.051) (Table 1).

### Pediatric-onset CD patients carrying *NOD2* variant alleles display a higher disease activity and underweight

**Figure 2:**
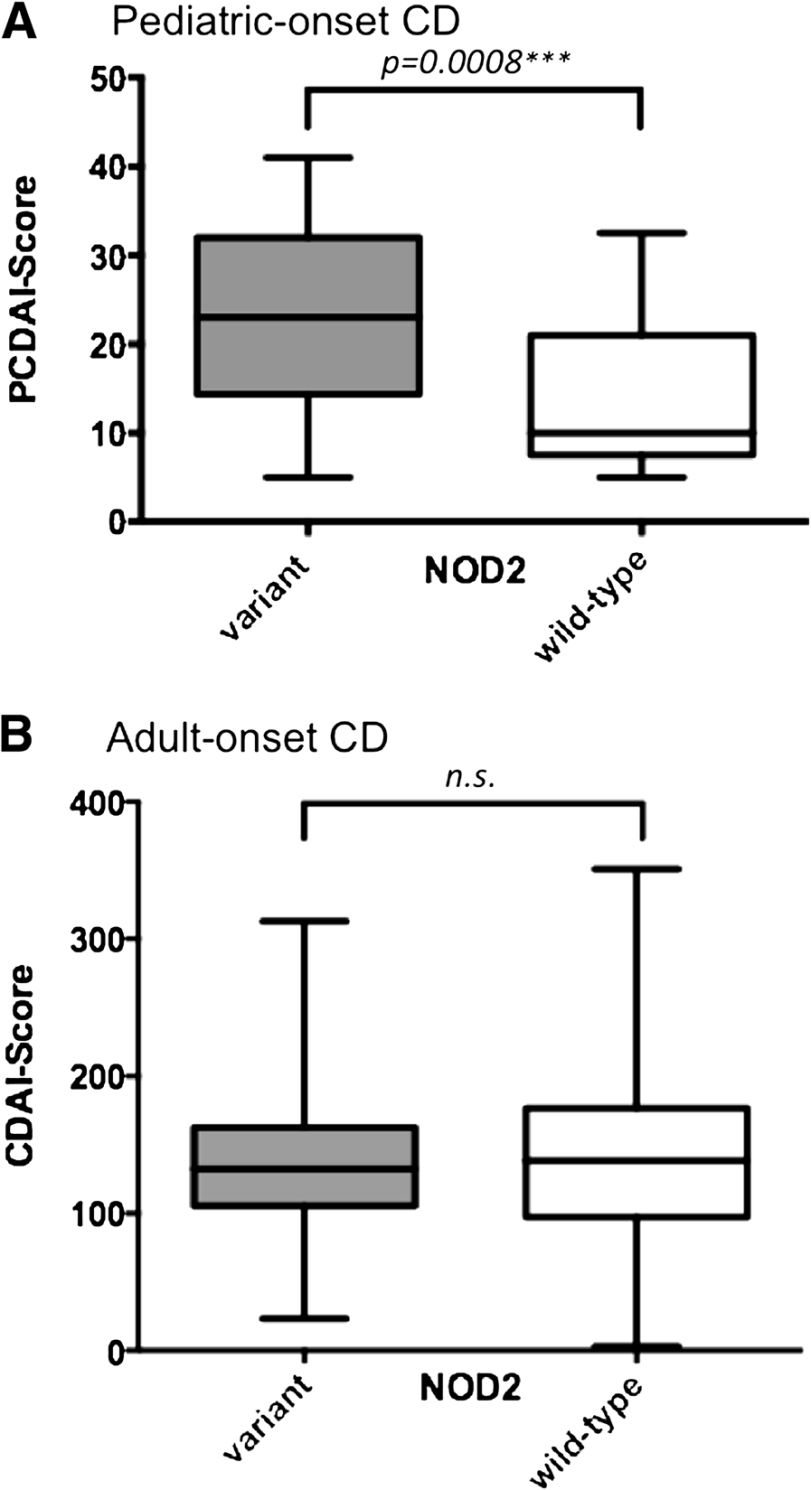
**Effect of *NOD2 *variants on PCDAI and CDAI Score in pediatric- and adult-onset CD patients.** CD patients with pediatric-onset (**A**) and adult-onset (**B**) are compared regarding *NOD2* variant (grey bars) or *NOD2* wild-type alleles (white bars) and their average PCDAI or CDAI Score. The median (lines within the boxes), the interquartile range (boxes) and the whole range are given. Differences were compared using unpaired, two sided Mann–Whitney test. A p-value below ≤0.05 was considered to be significant.

There is no significant influence of the main *NOD2* variants in patients with CD towards extraintestinal manifestation, need for surgery or complications (Figure 1). However, we found chronic active or high active course of disease in CD patients associated with pediatric-onset of disease (p=0.0001) and any of the main *NOD2* variant alleles (p=0.0001). The average PCDAI-Score over time was significantly higher in pediatric-onset CD patients with at least one *NOD2* variant than two wild-type alleles (23.2 versus 14.1; p=0.0008) (Figure 2A), and a chronic active (PCDAI≥10 or CDAI≥150; p=0.017) or moderate to severe course (PCDAI ≥ 30 or CDAI≥220; p=0.027) of disease significantly more frequent. However, in the adult-onset group disease activity and moderate to severe course measured by CDAI was independent of the *NOD2* genotype (Figure 2B).

**Figure 3:**
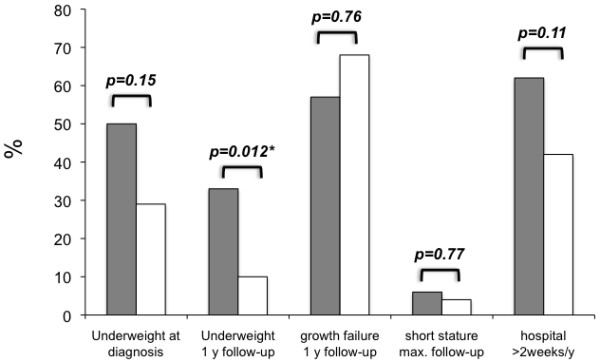
**Effect of NOD2 variants on underweight, growth failure, short stature and hospitalization in pediatric-onset CD patients.** Pediatric-onset CD patients with *NOD2* variant (grey bars) or *NOD2* wild-type alleles (white bars) were compared regarding underweight at diagnosis and during follow up, growth failure, short stature and hospitalization for more than two weeks per year. BMI at diagnosis and growth delay at one-year follow-up was available from 51 out of 85 patients (39% *NOD2* variant allele carriers). Differences were compared using chi-square or Fishers exact test, respectively. A p-value below ≤0.05 was considered to be significant.

Pediatric-onset CD is associated with growth delay at diagnosis and during the first year after diagnosis, however only underweight during course of disease is associated with patients carrying *NOD2* variant alleles (p=0.012; Figure 3).

### Osteoporosis is associated with *NOD2* variants, inflammatory course of disease and underweight

**Figure 4:**
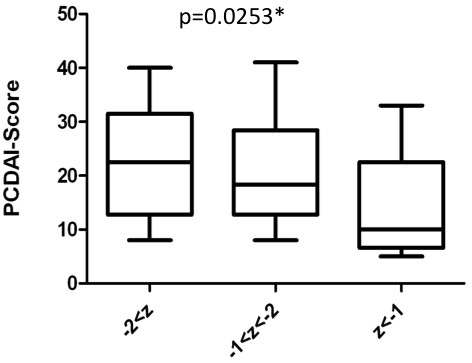
**Effect of PCDAI on BMD.** CD patients with pediatric-onset are compared regarding their PCDAI and BMD z-value. The median (lines within the boxes), the interquartile range (boxes) and the whole range are given. Differences were compared using Kruskal-Wallis test. The medians varies significant (*), p-value is 0.0253 using Gaussian approximation.

BMD measured by DEXA was available for 77 pediatric- and 91 adult-onset CD patients. Osteoporosis was associated with patients carrying *NOD2* variant alleles (p=0.033) and pediatric-onset of CD (p=0.022). Multivariate analysis demonstrated a 2.4 times increased risk for osteoporosis in CD patients carrying any *NOD2* variant allele (p=0.02). There was also a suggestive trend for CD patients with *NOD2* variant alleles and pediatric-onset towards osteoporosis (p=0.12, Figure 1; and by multivariate analysis p=0.07) and low BMD (Z-score below −1 p=0.10; Table 2). Especially pediatric onset CD patients with homozygous *NOD2* variant alleles are prone to osteoporosis and osteopenia (p=0.037; Table 2) and lower Z-scores respectively (p=0.033). We further evaluated the impact of factors independent of the NOD2 genotype influencing BMD. 46 out of 77 patients had a low BMD (Z-score below −1) thereof 23 (30%) suffered from osteoporosis (Z-score below −2.0) and 23 (30%) from osteopenia, only 31 (40%) had normal BMD (Table 2). The average disease duration was around 12 years in the low and normal BMD group. Osteoporosis at diagnosis of CD or in the follow-up was not associated with surgery, complications, extraintestinal manifestation or ileal or ileocolonic involvement (Table 2 and data not shown). However, patients with low BMD Z-scores had a significantly higher PCDAI-score (p=0.0092) (Figure 4). A multivariate regression model demonstrated that the PCDAI-score is 5.8 points higher in patients with low BMD than normal BMD, whereas this effect is adjusted for steroid dependent or refractory course of disease and underweight (p=0.03). In addition, underweight at diagnosis (p=0.045) and after 1year of follow up (p=0.011) but not short stature was significant associated with low BMD (Table 2). The need for immunosuppressive treatment with azathioprin (AZA) or methotrexate (MTX) was associated with low BMD (p=0.016) and there was a trend to steroid-refractory or dependent course (p=0.1) or anti-TNFα therapy (p=0.09). Interestingly, 57% of the patients with low BMD were steroid- or immunosuppressiva naïve at the time of the first measurement (data not shown).

**Table 2:** Characteristics of 77 Crohn patients with pediatric-onset according to the Montreal classification regarding BMD adjusted for age

**Pediatric-onset CD patients**	**Low BMD**	**Normal BMD**	**p-value**
	**Z-score<−1**	**Z-score ≥−1**	
**n=77**	**n=46**	**n=31**	
**%**	**60%**	**40%**	
***NOD2 *variant n (%)**	**24 (52%)**	**10 (32%)**	p=0.10
One variant allele	17	5	p=0.061
Two variant alleles	7	5	p=0.75
Homozygous variant alleles	7	0	p=0.037*
**Gender: female/male in %**	**45/55**	**48/52**	p=0.89
**Age: y**			
mean/SD	25/12.8	24/13.9	p=0.62
median/interquartile range	21/17-30	19/15-29	p=0.29
average disease duration/SD	12.2/11.6	11.6/10.8	p=0.84
**Age at diagnosis: y**			
mean/SD	13/3.6	12/5.1	p=0.33
median/interquartile range	14/11-16	13/10-17	p=0.69
**Localization: n (%)**			
L1 ileal	8 (17%)	9 (29%)	p=0.27
L2 colonic	8 (17%)	6 (19%)	p=1.0
L3 ileocolonic	30 (65%)	16 (52%)	p=0.25
L4 isolated upper disease	0 (0%)	0 (0%)	p=1.0
Upper GI involvement	15 (33%)	13 (42%)	p=0.47
**Behaviour: n (%)**			
B1 non-stricturing/penetrating	27 (59%)	20 (65%)	p=0.64
B2 stricturing	11 (23%)	6 (19%)	p=0.78
B3 penetrating intern	6 (13%)	3 (10%)	p=0.73
B3p penetrating perianal	9 (19%)	3 (10%)	p=0.34
Underweight at diagnosis	14/27 (52%)	5/22 (32%)	p=0.0453*
Underweight in 1y follow-up	14 (30%)	2 (6%)	p=0.0113*
Short stature	5 (11%)	1 (3%)	p=0.39
**Treatment: n (%)**			
Steroid dependent/refractory	23 (50%)	9 (29%)	p=0.10
Azathioprine / MTX	35 (76%)	15 (48%)	p=0.016*
Anti-TNFα-antibody	20 (44%)	7 (26%)	p=0.09

* indicates p value ≤ 0.05 considered to be significant.

### Influence of *NOD2* genotype on therapy regarding age at diagnosis of CD

**Figure 5:**
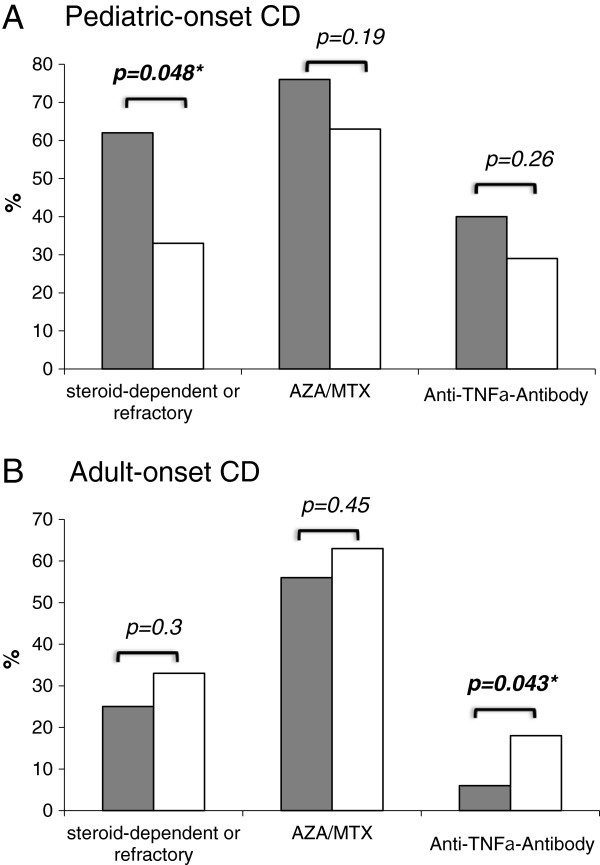
**Effect of *NOD2 *variant alleles on treatment in pediatric- and adult-onset CD.** CD patients with pediatric-onset (**A**) or adult-onset (**B**) of disease with *NOD2* variant (grey bars) or *NOD2* wild-type alleles (white bars) are compared regarding steroid-dependent or refractory course of disease, need for long-term immunosuppressive therapy with azathioprine (AZA) and methotrexate (MTX) or anti-TNFα antibody. Differences were compared using chi-square or Fishers exact test, respectively. A p-value below ≤0.05 was considered to be significant.

Comparing therapy and *NOD2* genotype revealed divergent trends between pediatric- and adult onset CD. Steroid dependent or refractory course of disease was more frequent in pediatric-onset CD patients with *NOD2* variants (p=0.048) (Figure 5). In addition, the use of long-term immunosuppressive drugs such as anti-TNFα agents; azathioprine or methotrexate trend to be higher in patients with NOD2 variant alleles and pediatric onset in contrast to adult-onset CD (Figure 5). This is also confirmed by analyzing the relationship between age of diagnosis, response to therapy and *NOD2* genotype. A significant age dependent difference towards therapy was only found in CD patients carrying *NOD2* variants. CD patients carrying at least one *NOD2* variant allele and being young at diagnosis (median age below 20 years) are at risk for steroid dependent or refractory course of disease (p=0.0022), long-term immunosuppressive therapy with AZA or MTX (p=0.0426) and the use of anti-TNFα agents (p=0.0004) (Additional file 1: Figure S1). In addition, carrying a NOD2 variant allele is associated with a 3.1 increased risk for steroid dependent or refractory course of disease (p=0.02).

## Discussion

Evaluation of disease behaviour by genotype is complex and may change throughout the course of disease. Dissecting pediatric- and adult-onset CD represents a model to identify further associations between *NOD2* gene variants and phenotypes and may help in selecting individual treatment regimes for patients with CD. We here demonstrate greater influence of *NOD2* variants in pediatric-onset CD patients towards higher disease activity, risk for underweight, osteoporosis and more intensive immunosuppressive therapy. This is the first study identifying an influence of *NOD2* variant alleles in patients with CD on osteoporosis and an age of onset dependent risk for steroid dependent or refractory course, long-term use of immunosuppressive drugs.

In this retrospective single center study in a tertiary center we report a high prevalence of CD patients carrying at least one *NOD2* variant allele with 44% in the pediatric- and 42% in adult-onset CD similar to other European pediatric multicenter cohort studies with a prevalence of 35 up to 45.6% [12,23,24]. In addition, the presence of two *NOD2* mutant alleles was found more frequent in the pediatric-onset CD patients as reported by others [23,25] and may predict complicated disease [26].

Growth retardation is a frequent specific feature of pediatric-onset CD [27], which manifest as poor weight gain or short stature and may be the first presenting symptom. We observed a trend in CD patients with *NOD2* variants towards underweight at diagnosis in our pediatric-onset cohort analogous to other studies [28-30] describing lower weight and a tendency to growth retardation more or less pronounced in their pediatric cohorts. In addition, we found also significant ongoing underweight in the course of disease in patients with *NOD2* variant alleles. Growth failure seems to be associated with disease severity [29] and high levels of TNFα in pediatric IBD [31]. Indeed, the average disease activity in pediatric-onset CD patients carrying at least one *NOD2* variant allele was significantly higher and associated with chronic active or moderate to severe clinical course of disease. However, other studies did not found an influence of *NOD2* variants towards higher disease activity [25,29]. This is explicable by the fact, that in most instances disease activity was educed from hospitalization, need for immunosuppressive drugs, surgery or occurrence of complications but not by PCDAI at onset and during course of disease [25,28,29,32]. The PCDAI-score was only used by one other pediatric study with 65 CD patients, which described an influence of *NOD2* variants towards a higher PCDAI [30]. Besides actual history and laboratory values (e.g. haematocrit, erythrocyte sedimentation rate and albumin) the PCDAI score includes also weight loss, growth delay, perirectal disease and extraintestinal manifestation. Therefore a high PCDAI score in our pediatric-onset CD patients with *NOD2* variants reflects a more severe disease behaviour indicated by underweight, stricturing and penetrating behaviour. Moreover, an aggressive disease manifestation in pediatric-onset CD patients with *NOD2* variants leading to complications and surgical resection is described by Lacher et al. [12] and Kugathasan et al. [32,33].

The prevalence of osteoporosis ranges from 7 to 30% and osteopenia from 22 to 70% in pediatric IBD studies [13,34,35]. In fact, 30% of our patients with pediatric-onset CD have osteoporosis and 31% osteopenia, some even before corticosteroid treatment confirming data from Walther [13] and Harpavat et al. [36]. Possible risk factors for pathologic bone density in CD are disease activity, lifetime steroid dosage over 10 g, CD, multiple bowel resections, young age and low body mass index according to a large epidemiological study [37]. We also observed low BMI and a higher disease activity associated with reduced bone density in pediatric-onset CD. Similar Paganelli et al. described an inversely correlation of volumetric BMD with disease activity measured by PCDAI and IL-6 [38]. Other risk factors such as ileal localization and bowel resection did not play a role in our pediatric-onset cohort. Lifetime corticosteroid treatment in children with IBD seems to be less important as previously believed, as children with a chronic non-inflammatory type of disease such as steroid sensitive nephritic syndrome (SSNS) receiving glucocorticoids chronically do have a better bone mineral density than children with CD [39]. Certainly osteoporosis was associated with a steroid refractory or dependent course of disease in the pediatric-onset group. In fact the glucocorticoid dosage in the treatment of pediatric CD may be not as high as in previous older studies; children with CD often receive enteral nutrition therapy and there are more patients with steroid dependent or refractory course of disease in the low BMD group actually switched earlier to a long-term immunosuppressive therapy including azathioprine, methotrexate and anti-TNF-α antibody treatment. Thus the inflammatory state in CD might basically contribute to high-turn over bone mineral loss. It is also known that *NOD2* variant alleles induce an inflammatory response e.g. via NF-кB activation [40]. Thus some cytokines with osteolytic activity such as TNFα, IL-1β, IL-6, IFNγ, RANK, RANKL and osteoprotegrin may also be elevated. IL-1*β* for example stimulates osteoclast activity and enhances mineral bone loss. In fact carriers of *IL1β -511b* allele have significant lower BMD [41]. Interestingly, CD patients carrying *NOD2* variant alleles have an increased risk towards osteoporosis. This is more pronounced in pediatric-onset CD, especially in patients carrying homozygous *NOD2* variant alleles. However, we could not prove our hypothesis that osteoporosis is significantly more frequent in pediatric-onset CD patients carrying any *NOD2* variant allele may be due to a lack of power. Taken together, these data support screening of osteoporosis in pediatric onset CD, especially in children with *NOD2* variants, low BMI and high PCDAI.

We recently reported that CD patients with *NOD2* carrier status were more refractory for steroid treatment but responded well to immunomodulators such as AZA/6-MP [42]. This was in contrast to another study that could not find an association between *NOD2* carrier status or age of onset of disease and response to steroids [43]. We speculated that treatment response might vary analyzing pediatric and adult CD patient study cohorts regarding *NOD2* genotype. To corroborate this hypothesis we now included a pediatric CD cohort and reclassified all CD patients in our adult cohort according to the onset of disease in pediatric and adult onset. Using this approach we found a significant association for steroid failure in CD patients with *NOD2* variant alleles and younger age of onset in contrast to Weiss et al. [43]. In addition, the need for long-term immunosuppressive therapy and second line therapy with anti-TNF-alpha agents was also only associated with younger age of onset and the presence of any *NOD2* variant allele. Although a positive response to the anti-TNFα agent infliximab was found to be associated with a higher systemic inflammation [44], the *NOD2* genotype was not predictive of treatment outcome in their cohort [45]. The diverging results regarding our study cohort might be due to the subgroup analysis of pediatric-onset CD. The fact that we did not investigate treatment outcome is another disadvantage of this study and its retrospective approach.

## Conclusion

In summary, dissecting a large unselected group of patients with long-term CD duration in pediatric- and adult-onset of disease revealed a more pronounced difference between *NOD2* genotype and phenotype in the pediatric-onset group. There was an association of *NOD2* gene variants towards a more affected phenotype with underweight, higher disease activity, steroid failure and the need for long-term and intensive immunosuppressive therapy. Thus pediatric-onset CD and *NOD2* variant alleles are risk factors frequently associated with chronic and moderate to severe course of disease. In addition, the prevalence of low bone mineral density is considerably high in pediatric Crohn patients with high average PCDAI, low BMI, steroid failure and *NOD2* variant alleles.

The major goal in the treatment of CD is to change the fatal course of chronic active disease especially in children and adolescents in order to improve growth and prevent osteoporosis. Until now testing for *NOD2* variants is not part of the diagnostic work-up in pediatric-onset CD. We conclude from our study, that genetic testing for *NOD2* variant alleles is useful in pediatric-onset CD as it may identify patients who are at risk for a more affected phenotype. Given the beneficial effect of TNF-α blockade on bone formation and on CD activity this may be an alternative early treatment strategy in children [5,46].

## Abbreviations

6-MP: 6-Mercaptopurine; AZA: Azathioprine; BMD: Bone mineral density; CARD 15: Caspase-activation recruitment domains 15; CD: Crohn’s disease; CDAI: Crohn’s disease activity index; IBD: Inflammatory bowel disease; SNP: Single nucleotide polymorphism; NOD2: Nucleotide oligomeric domain 2; PCDAI: Pediatric Crohn’s activity index; SNP: Single-nucleotide polymorphism; TNF: Tumor necrosis factor; UC: Ulcerative colitis; WT: Wild-type.

## Competing interest

There is no potential conflict of interest of any author relevant to the manuscript.

## Authors’ contributions

CP conceived of the study, participated in the coordination and collection of data, performed the statistical analysis and drafted the manuscript. GL carried out the molecular genetic studies. VP, JHN and JK carried out the coordination and data collection. BM performed the statistical analysis. GvB and KMD participated in the design and coordination of the study. All authors read and approved the final manuscript.

## Pre-publication history

The pre-publication history for this paper can be accessed here:

http://www.biomedcentral.com/1471-230X/13/77/prepub

## Supplementary Material

Additional file 1: Figure S1 Effect of age of onset at diagnosis of CD and NOD2 variants on treatment. Th age of onset of CD in 202 patient with NOD2 variant (grey bars) or wild-type (white bars) alleles is compared regarding steroid-dependent or refractory course of disease (A), need for long-term immunosuppressive therapy with azathioprine (AZA) and methotrexate (MTX) (B) or anti-TNFɑ agents (C). Differences were compared using Kruskal-Wallis test. A p-value below ≤0.05 was considered to be significant and indicated with an asterix. Click here for file
