# Supplementary figures and images for: Age-of-onset-dependent influence of NOD2 gene variants on disease behaviour and treatment in Crohn’s disease
Source: BMC Gastroenterol. 2013 May 2;13:77. doi: 10.1186/1471-230X-13-77 (PMC3659055; doi:10.1186/1471-230X-13-77)

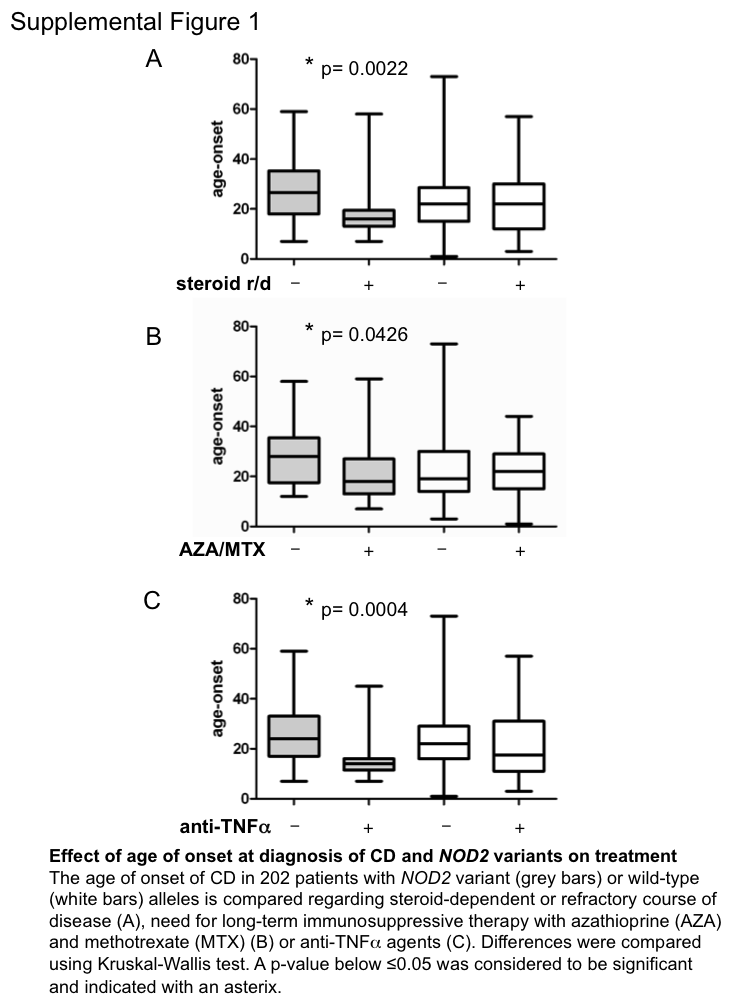

Supplement: Additional file 1: Figure S1 — Effect of age of onset at diagnosis of CD and NOD2 variants on treatment. Th age of onset of CD in 202 patient with NOD2 variant (grey bars) or wild-type (white bars) alleles is compared regarding steroid-dependent or refractory course of disease (A), need for long-term immunosuppressive therapy with azathioprine (AZA) and methotrexate (MTX) (B) or anti-TNFɑ agents (C). Differences were compared using Kruskal-Wallis test. A p-value below ≤0.05 was considered to be significant and indicated with an asterix. [file 1471-230X-13-77-S1.tiff]
